# Learning Region-Based Attention Network for Traffic Sign Recognition

**DOI:** 10.3390/s21030686

**Published:** 2021-01-20

**Authors:** Ke Zhou, Yufei Zhan, Dongmei Fu

**Affiliations:** 1 Collaborative Innovation Center of Steel Technology, University of Science and Technology, Beijing 100083, China; zhouke@ustb.edu.cn; 2 School of Advanced Engineering, University of Science and Technology, Beijing 100083, China; 41718057@xs.ustb.edu.cn; 3 School of Automation and Electrical Engineering, University of Science and Technology, Beijing 100083, China

**Keywords:** traffic sign classification, attention, region-based, ice environment, ice traffic sign, recognition benchmark, ice traffic sign detection benchmark

## Abstract

Traffic sign recognition in poor environments has always been a challenge in self-driving. Although a few works have achieved good results in the field of traffic sign recognition, there is currently a lack of traffic sign benchmarks containing many complex factors and a robust network. In this paper, we propose an ice environment traffic sign recognition benchmark (ITSRB) and detection benchmark (ITSDB), marked in the COCO2017 format. The benchmarks include 5806 images with 43,290 traffic sign instances with different climate, light, time, and occlusion conditions. Second, we tested the robustness of the Libra-RCNN and HRNetv2p on the ITSDB compared with Faster-RCNN. The Libra-RCNN performed well and proved that our ITSDB dataset did increase the challenge in this task. Third, we propose an attention network based on high-resolution traffic sign classification (PFANet), and conduct ablation research on the design parallel fusion attention module. Experiments show that our representation reached 93.57% accuracy in ITSRB, and performed as well as the newest and most effective networks in the German traffic sign recognition dataset (GTSRB).

## 1. Introduction

The development of a high-precision automatic traffic sign recognition systems is an important subject and is of great significance for self-driving and modern driving assistance technologies. A traffic sign recognition system has two stages, detecting and then classifying traffic signs, however, the traffic sign recognition (TSR) problem is typically thought to be traffic sign classification, regarding how to classify traffic signs accurately, some of which are quite similar in a large amount of categories.

Research for the detection and recognition of traffic signs began as early as Paclik’s research in 1984. Several traffic sign detection and recognition datasets have been proposed, including the German traffic sign detection and recognition dataset (GTSDB and GTSRB) [1,2], which is the most popular dataset used in TSR research; Belgium Traffic Sign Dataset (BTSD) [3]; Sweden Traffic Sign Detection Dataset STSD [4]; and Tsinghua-Tencent 100 K [5], which is one of the biggest realistic street photo datasets with many smaller signs and so on. Based on the GTSRB and GTSDB, an International Joint Conference on Neural Networks (IJCNN) competition was held to encourage researchers to come up with a more accurate method for TSR. The methods proposed before can be divided into two categories, the traditional feature extraction and classifier design methods and deep-learning methods.

Traditional methods [6,7,8,9] use dimension reduction methods, like principal component analysis and the Karhunen–Loève transform, to extract effective and lower-dimension features and select a corresponding classification method, like Fisher, support vector machines (SVM), and multi-layer perceptron (MLP). Traditional methods require a smaller dataset and less computation; however, the accuracy relies on the data distribution and is relatively lower. Deep-learning methods [10,11,12,13,14] employ neural networks to extract features and loss functions and optimization algorithms to train a great classifier. Deep-learning methods typically require more data and better computation machines, such as a GPU, to train a model, and can obtain a higher accuracy. Many research teams began to focus on the frontier of traffic sign recognition including the occlusion problem, angle problem, and small targets, and to design specific networks for this.

However, the application of automatic classification technology for traffic signs in harsh environments, such as ice and snow, is still insufficient. Due to the complexity and diversity of the weather and lighting conditions, the harsh environment of winter driving still makes related computer vision more difficult. Problems, such as the size and occlusion of traffic signs in environments, makes it more difficult to obtain information from the pictures, and presents greater challenges in the detection and classification of traffic signs. A Russian team [15] published a Russian ice and snow environment automatic driving dataset called IceVisionSet containing incomplete traffic sign annotations; however, the research focused on the light effects on a traffic sign recognition model in ice and snow environments and did not propose a useful model.

Currently, few teams have used advanced object detection and classification models to conduct research and applications on this dataset, and the dataset also requires processing. At the same time, the traditional method of simultaneous detection and classification is often not effective in such cases. In this article, our research goal is to achieve a better classification accuracy in the ice and snow dataset based on [15] through our design. The contribution points of this article are as follows:

According to the traffic pictures collected under the ice and snow environment of [15], we produced a more challenging ice and snow environment traffic sign detection dataset (ice environment traffic sign detection benchmark (ITSDB)) and a snow and ice environment traffic sign classification dataset (ice environment traffic sign recognition benchmark (ITSRB)) that both contain different sizes and complex environmental factors:

(1) We transplanted and trained the two newest detection networks, HRNetv2p [16] and Libra-RCNN [17], and evaluated them on the ice and snow dataset, ITSDB, to verify the robustness of the network and the challenges our dataset proposes.
(2) Based on the attention mechanism, we designed two types of attention modules and a new type of traffic sign classification network called PFANet overcoming the complex factors of the ice and snow environment, and we achieved the state-of-the-art 93.570% accuracy with the ITSRB and verified its robustness on the public dataset.

The paper is organized into several sections. In Section 2, related works about classification and detection and their application in traffic signs and the attention mechanisms applied in these tasks is presented. Section 3 introduces two traffic sign datasets and the traffic sign classification method with the attention mechanism we proposed. Section 4 presents the performance of some of the most advanced detection methods in our detection dataset, and the experimental results of our method, including the comparison study and ablation study. Finally, Section 4 is the discussion about the experiment results, and Section 5 is our conclusions for the presented work.

## 2. Related Works

### 2.1. Classification of Traffic Signs

Before the improvement of the computation ability and the development of neural networks, methods using a combination of selected feature representation, dimension reduction, and classifiers were the most popular methods used in this topic. Han et al. [6] focused on the feature exaction and proposed an advance speeded-up robust features (SURF) algorithm with an extracting high priority matches selection strategy. Zaklouta et al. [7] compared the K-d trees with random forest using four types of histogram of oriented gradients (HOG) and distance transform and achieved the best accuracy with tree classifiers. Maldonado-Bascón et al. [8] trained a shape-based support vector machine (SVM) on the distance to border vectors, and Fleyeh et al. [9] proposed a two stage SVM method, with the first stage to classify the shape and the second stage to determine the pictogram.

Due to convolutional neural networks’ greater performance on large datasets, traditional methods based on SVM are gradually being eliminated, and recognition problems in special cases are gradually solved. Ciresan et al. [10] showed the better performance when combining the CNNs and MLP as a committee. Sermanet et al. [11] changed the classifier input with different scales of convolution features fed into the classifier instead of the only high-level features. Instead of concatenating the different scale features, Ciresan et al. [12] creatively added different branches of features after convolution together to enhance the single scale convolution performance. Wong et al. [13] proposed a highly compact deep convolutional neural network based on macroarchitecture design principles to decrease the network parameters and make the network small. Li et al. [14] described an Efficient CNN applying the Inception module to acquire different-level features and concatenated them to strengthen the feature representation.

Other networks researched specific problems in the TSR field. Zhu et al. [5] proposed the Tsinghua-Tecent100 K dataset for the problem of small traffic sign recognition and added a category recognition branch on the basis of [18] to classify small targets more accurately. Hou et al. [19] adopted the 1 v 1 method for blocked traffic signs, based on the HOG feature of the block, and calculated the confidence level for classification. Khan et al. [20] designed the dark area sensitive mapping (DASTM) technique to improve the detector’s effect in low-light traffic sign recognition, reaching 100% in GTSDB.

### 2.2. Attention Mechanism and Applications in TSR

The attention mechanism was originally used to solve the word order problem of machine translation and the problem of information association at the beginning of sentences. In RNN and seq-to-seq, Bahdanau et al. [21] calculated the attention weight, Wa, and optimized training through the hidden layer state and alignment of the input and output sequences, and achieved good results. Subsequently, the attention model began to be applied in computer vision, and achieved good results. Xu et al. [22] established a hard attention mechanism and a soft attention mechanism by designing attention to a single position and a weighted ϕ function, which effectively reduced the number of parameters and the calculation time of the traditional network leading an upsurge of attention mechanisms.

In 2015, Luong et al. [23] proposed an improved version of the global attention mechanism that considered all the hidden states of the encoder and a local attention mechanism that effectively reduced the amount of calculation and complexity. In view of the long training time needed for RNN and seq-to-seq, Vaswani et al. [24] proposed the transform model and self-attention mechanism to calculate the output and input without a RNN, that is, the introduction of queries, keys, values, and scaling the dot product method to calculate the attention weight. Comparing with operating on pixels, Hu et al. [25] achieved the distribution of attention on the channel through squeeze and excitation, and achieved the best results.

As it had shown great performance in other problems, researchers began to apply the attention mechanism to the TSR problem. Arcos-Garcı’a et al. [26] combined the spatial transform module and a classical CNN network and applied the spatial transform on the input and convolution procedure to increase the spatial invariance of CNNs. Uittenbogaard et al. [27] proposed a residual attention mechanism combined with dense concatenation and multi-scale discriminators to preserve background information to improve the classification ability. Zhang et al. [28] added a channel attention module acquiring global information after region of interest (RoI) pooling to improve the ability to recognize small traffic signs.

## 3. Materials and Methods

### 3.1. Benchmark

#### 3.1.1. Data Collection

Unlike most datasets, such as ImageNet and COCO, which collect pictures through online keyword indexing, or Tsinghua-Tencent 100 K, which collects pictures through Tencent Street View, the original pictures of our dataset were selected from the traffic photos marked IceVisonSet [15]. The picture of the sign lights and storage method is from street views of the real snow and ice season in Russia. Compared with the pictures collected separately, the pictures in our dataset are from continuous lossless video captured by interval sampling. Shooting through a 2 K camera in a car better mimics the perspective of a self-driving car. At the same time, through sports camera sampling, compared with fixed point and fixed distance sampling, the dataset contains unified signs at different distances and different angles, which is more challenging.

#### 3.1.2. ITSDB

ITSDB is the traffic sign detection dataset under environments, which is used for the task of traffic sign detection. The dataset contains pictures under various characteristics. Under the premise of environments, the dataset contains various pictures under different light conditions, time conditions, environmental factors, and occlusion conditions. At the same time, the dataset contains traffic signs of different sizes. According to the size of the signs, we divided the included traffic signs into small, medium, and large, as shown in Table 1.

**Figure 1:**
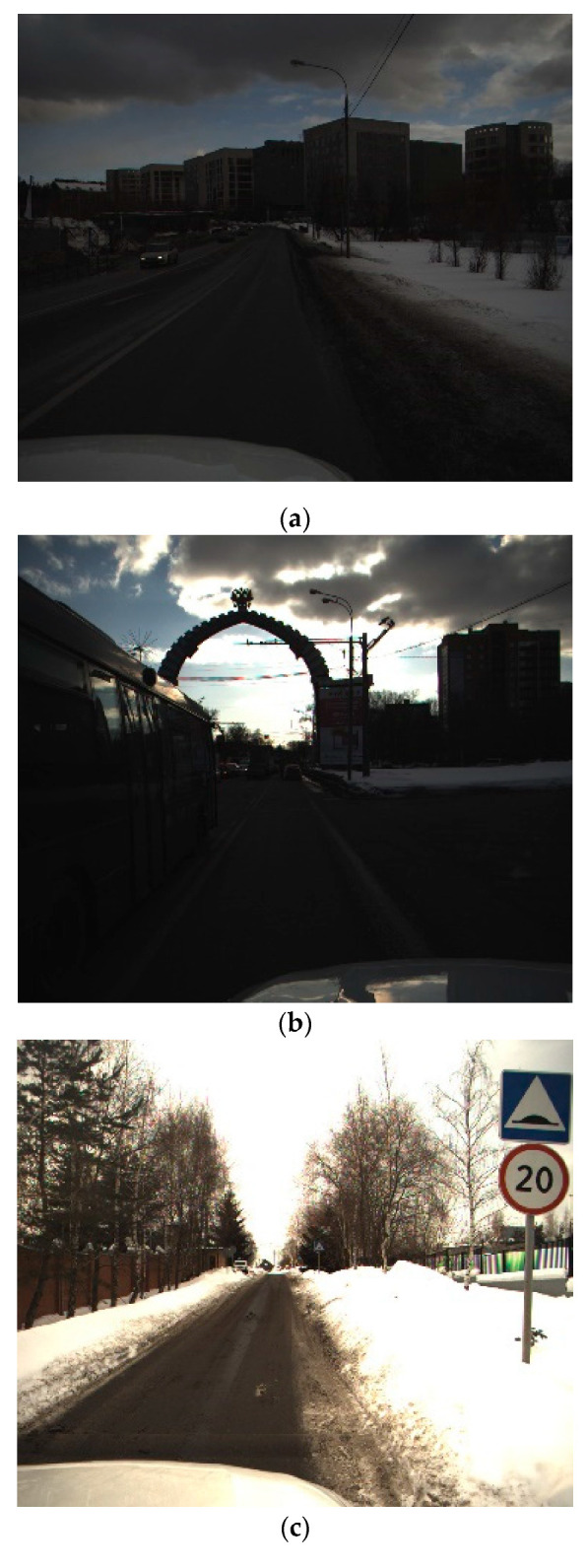

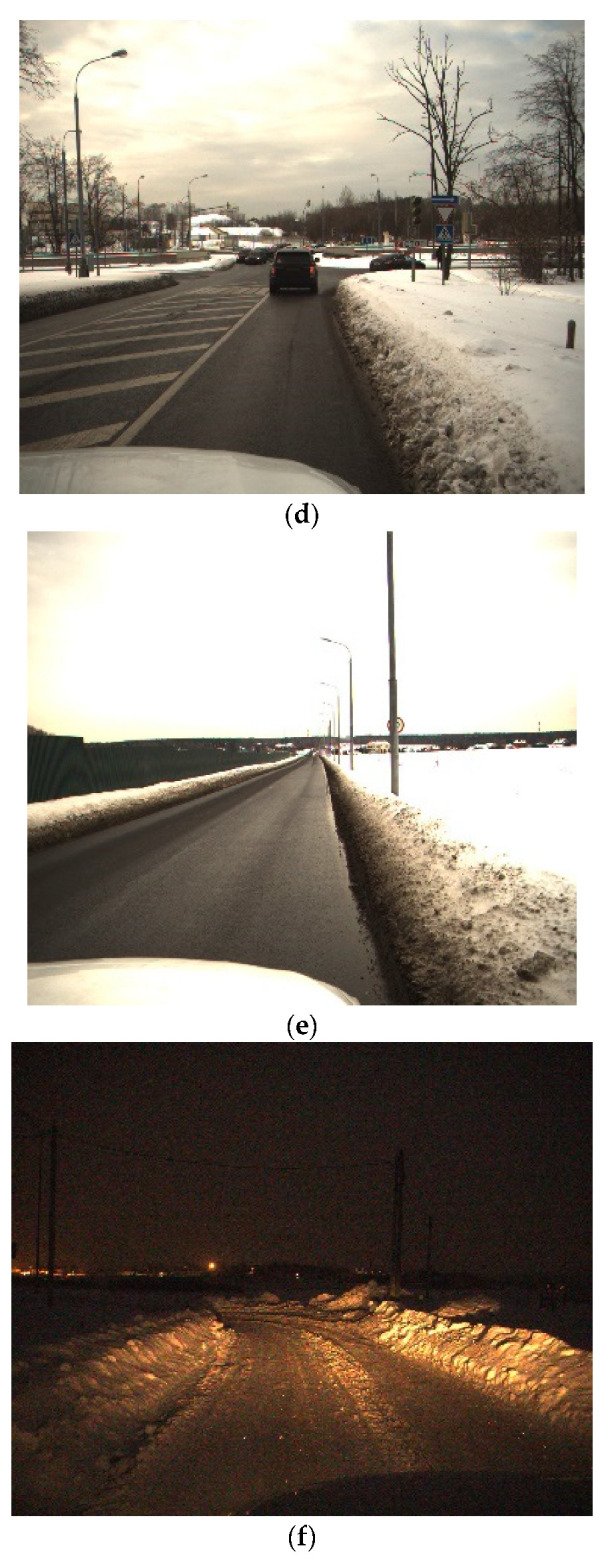
(**a**) Under an ice and snow environment with dark light. (**b**) Under an ice and snow environment with strong reflection. (**c**) Under an ice and snow environment with strong white light. (**d**) Normal. (**e**) Occlusion. (**f**) Night.

The ice and snow environment is not only just a representative environment, but also has a strong influence on the brightness, completeness, color saturation, and other information of the picture, which have been proved to have a great impact on traffic sign detection and classification [15,29]. The dataset includes these types of pictures and pictures of day and night, shown in Figure 1, which are more challenging for TSR problem not included in most public dataset like GTSRB. In addition, comparing with the most used public dataset GTSRB, our dataset has much higher percentage of small targets. If one model is trained and can achieve excellent results on this dataset, it may have a better ability to overcome the influence of the environment.

As for the processing of the pictures, we converted the lossless pictures stored in the “rgba” format and the front-end picture format FLIF, wrote a program to convert to the general JPEG format, resized the pictures compressed to 1/2 the original size, and restored the pictures according to the annotations. The dataset was also preprocessed to remove unmarked pictures stored in the cloud server.

The dataset was divided according to the ratio of 8:2, the training set contained a total of 5806 pictures, and the test set contained a total of 1452 pictures. The picture size was 2448 × 2048, and a total of 43,290 traffic signs were marked. The number of traffic signs contained in each picture is uncertain. By disrupting the order of the pictures, the timing information brought by the video frame collection is eliminated. Due to the three-level classification of Russian traffic signs such as (1.2.12), the number of partial categories is too small, and the sample imbalance leads to poor universality. We only considered the first-level categories and merged the lower-level categories to merge the traffic signs, and divided these into nine categories. We used the COCO2017 object detection annotation format for annotation. The dataset composition was divided into a picture folder consisting of training pictures and detection pictures according to the format and the corresponding annotation file in JSON format.

#### 3.1.3. ITSRB

ITSRB is the traffic sign recognition dataset under an ice and snow environment, which was used for the classification task of traffic signs. The dataset images are cropped from the image in the ISTDB with bounding boxes in the annotation, only including the traffic sign and, thus, containing all the influencing factors in ITSDB, which poses great challenges to the classification network. Each picture contains only one traffic sign. In addition to the traffic sign, each picture also contains some environmental background information near the traffic sign.

**Figure 2:**
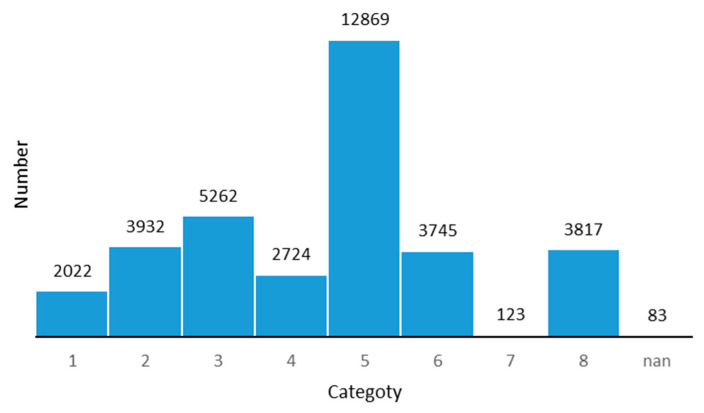
Data distribution in the train set of ice environment traffic sign recognition benchmark (ITSRB).

**Figure 3:**
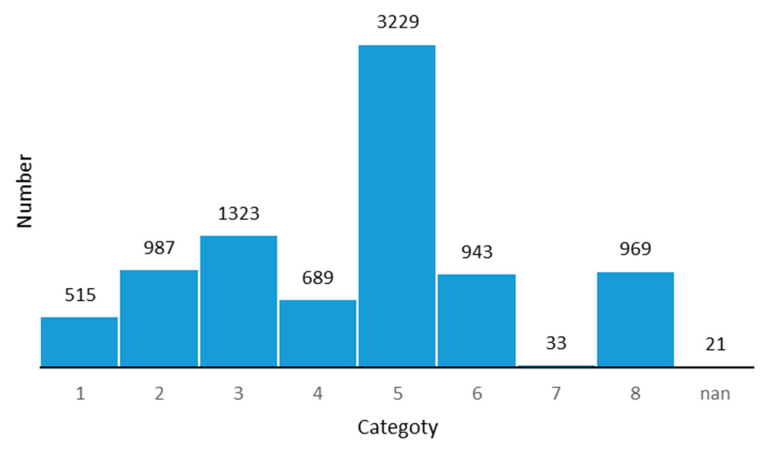
Data distribution in the test set of ITSRB.

The dataset was divided according to an 8:2 ratio, the training set contained 34,577 pictures, and the test set contained 8709 pictures. Pictures in the dataset were of different pixels as shown in Table 1. The original three-level classification method contained more than 130 categories, which is inconsistent with the number of samples and is prone to problems, such as non-convergence or oversaturation, and so that the training is of little significance. We mainly focused on the impact of the ice and snow environment on the data, and therefore we used the same merging method as the detection dataset, and obtained nine categories of traffic signs. The data distribution of the different categories is shown in Figure 2 and Figure 3.

### 3.2. Method

#### 3.2.1. Attention Module

First, we introduce the attention modules we designed in the PFANet. The two PFAN modules we designed were inspired by [24,30]. They inherited the scaled dot production attention with query, value, and key, and also have several innovations more suitable for this problem detailed below. With PFAN-A module, network can extract more information during feature extraction for classification, and PFAN-B module can gain more useful features during feature fusing between different resolution feature maps, both have been proved in our ablation study. Moreover, we propose combining both modules in the parallel structure network, which is also proved to achieve great performance in complicated ITSRB and GTSRB.

PFAN-A is a new type of self-attention. It takes only one feature map *C_f_* as input. By one convolution operation, we first find the query and key matrix, which will be used to generate the attention matrix later. Then, with the scaled dot operation, the attention weight matrix Tem is generated and is applied by a scaled dot operation again in the input, which can be thought as the value, to find the basic attention output. Finally, we add the basic attention output to the input to obtain the final attention output *H_f_*, which originated from the Resnet [31] and can improve the feature performance.

In the PFAN-A, different from W_k_ and W_a_ used to generate the weight matrix, W_v_ is used to extract features from the input and values, increasing the network depth. PFAN-B is a type of cross attention, and most operations are the same as PFAN-A. PFAN-B has two inputs, down-sampled *H_k_* as the query and key input, and *H_k+1_* as the key input. As *H_k_* has twice the resolution of *H_k+1_* and the scaled dot operation requires the same scale of inputs, a down-sample operation is applied in the *H_k_*. Then, the same operations are applied to obtain the final attention output *D_k+1_* of PFAN-B, in which W_v_ is still used to extract the features from the input and values, increasing the network depth.

**Figure 4:**
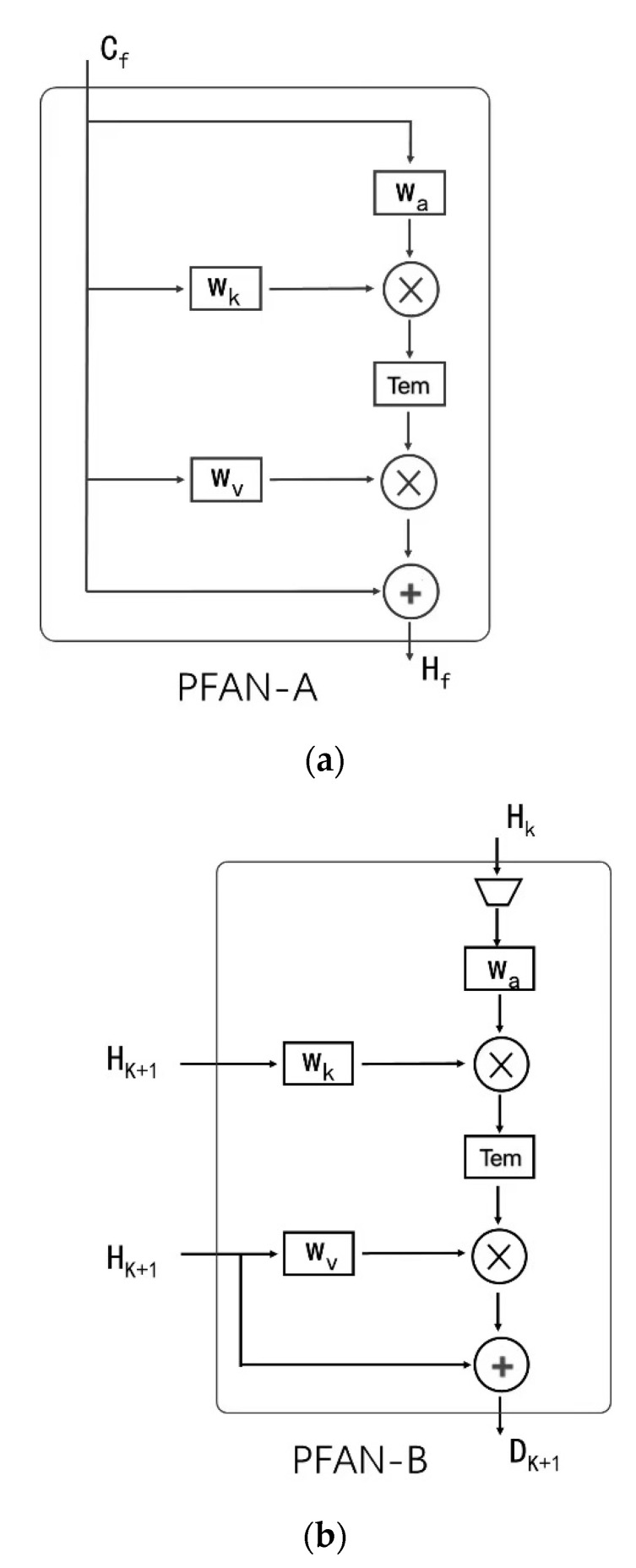
(**a**) PFAN-A block “
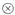
” denotes scaled matrix multiplication. “
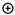
” denotes element-wise sum W_k_, W_v_, and W_q_. are 1 × 1 Convs with zero padding and the stride of 1. Tem denotes the scaled dot operation output. (**b**) PFAN-B block, “
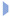
” denotes the down-sample operation, implemented by 2 × 2 MaxPooling.

Different from [24,29], in the PFAN-A structure (shown in Figure 4a), the query, value, and key are the same (such as C_1_, C_2_…C_4_ in Figure 4). We designed the structure to take advantage of the correlations of each part in the feature map at a resolution, to find and highlight features with high correlation with the classification category, and to give higher weights. For the design of the PFAN-B structure (shown in Figure 4b), we considered the characteristics of high-resolution preserving networks to extract features of different sizes, input different semantic and category information extracted from feature maps of different sizes into PFAN-B, and output *H_k_* query by the hidden layer with high resolution, using the value and key of the output *H_k+1_* from the low-resolution hidden layer to query and highlight the features related to classification in the feature maps of different resolutions. Both designs have been shown to have significant effects in ablation experiments.

#### 3.2.2. Parallel Fusion Attention Network

**Figure 5:**
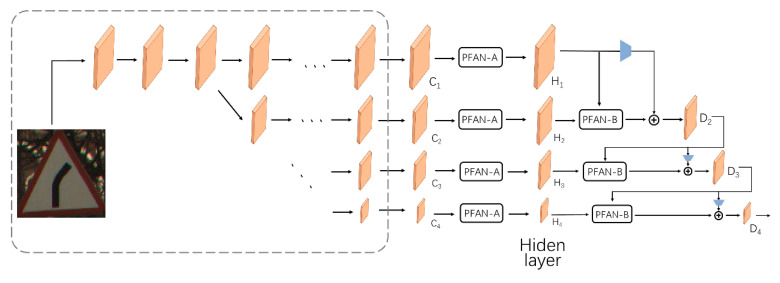
The framework of the PFAN for image classification we propose. Once {C_1_, C_2_, C_3_, C_4_} has been obtained, we further generate the hidden layer *H_f_*, and then *D*_1_, *D*_2_... progressively. “
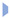
” denotes down-sampling implemented by 2 × 2 MaxPooling and “
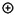
” denotes an element-wise sum. In the dashed rectangle, the horizontal arrow represents the bottleneck or the basic block operation, and the diagonally downward arrow indicates down-sampling implemented by 2 × 2 MaxPooling. Arrows outside the rectangle only represent the data flow direction.

An overview of our architecture is shown in Figure 5. The architecture includes a down-sampling parallel network that maintains high resolution (including HRNet and a parallel convolution to extract features) and three fusion attention modules. The input is a 3 × 224 × 224 picture. The output is Dn belonging to the space Rc×1, and c is the category of the dataset. The output is a nine-dimension tensor, with each representing the possibility of belonging to this category. Before the network computation, data preprocessing will be operated on the image, including the normalization and resizing.

The PFANet consists of two parts, the parallel feature extraction part and parallel fusion attention part. For the parallel feature extraction part, we employed the HRNet [16] to extract four feature maps of different scales. HRNet is well known for its high performance in human pose estimation, as well as object detection, classification, and instance segmentation. Different from traditional feature extraction method relying on a high-to-low and low-to-high framework or continuously down-sampling, HRNet keeps the high-to-low produced subnet in parallel in the network instead of in series.

With repeated multi-scale fusion between high-resolution and low-resolution representations, more information is obtained, increasing the accuracy. For our topic, it is essential to obtain richer information from limited resolutions. HRNet-w18 was employed to extract the features of the images shown in the dashed rectangle for the following attention and fusion operation. There are four stages increasing the branches from 1 to 4, the channels of which are 32, 64, 128, and 256, and the size of the feature map on nth branch is (224 × 224)/2^n−1^. The first two stages only have one module, and the last two have four and three modules.

Each module is composed of two blocks, and there are two kind of blocks, bottleneck and basic block, as designed in [30]. In the first stage, all the blocks are bottleneck, and the other stages use the basic block. Multi-scale fusion is operated between modules in each stage but is omitted in Figure 5 for simplification. After the different resolution extraction, four sizes of resolution feature maps are resized to fixed channels by convolution for further feature extraction, highlight, and fusion.

After acquiring the four feature maps, to help the parallel classification network extract more spatial information and better output results, we designed the parallel fusion attention module. The module contains input *C_f_* extracted by HRNet, hidden layer output H_m_, and output D^n^. First, PFAN-A is operated on each input *C_f_* to highlight the features related to the classification in different resolution feature maps, and the hidden layer output *H_f_* is obtained. The PFAN-A operation is performed on *C_f_* only once. Then, from the top to the bottom, every two adjacent hidden layer outputs *H_k_* and *H_k+1_* are used as the input of PFAN-B to obtain a feature map with the cross attention.

Specifically, *H_k_* is used as a query in the PFAN-B module to find the effective classification features in *H_k_*_+1_. After this, we add the down-sampled input *H_k_* to the output of PFAN-B, and find the final output of these two branches. This operation is repeated three times, decreasing the branches to 1. When the branch is waiting for the PFAN-B operation with the previous branch, one convolution operation is applied in this branch to maintain the network depth. The working process of this module is shown below. With more comprehensive and accurate spatial features, a fusion attention mechanism that combines multi-resolution features significantly improves the accuracy of classification prediction:

(1) $$H_{f}=\varphi_{1}\left(C_{f}\right).$$

(2) $$D_{k+1}=F\left(H_{k}\right)+\varphi_{2}\left(H_{k},\;H_{k+1}\right).$$

Among them, $\varphi_{1}\left(*\right)$ represents the PFAN-A operation, $\varphi_{2}\left(*\right)$ represents the PFAN-B operation, and F(∗) represents the down sampling operation, implemented with MaxPooling.

## 4. Results

### 4.1. Evaluation Metrics

In this paper, to evaluate and compare the performance of the advanced detection method in ITSDB comprehensively with different sizes of targets and average performance, the metrics (3–8) given below were used. In the comparison between the proposed method and other classification models, accuracy is employed to evaluate their performance, defined in metric (9).

Before defining the metrics used in the experiments, some basic metrics are defined below:

- *IoU* is the ratio of intersection and union of the predicted bounding box and the true bounding box. The mathematic form is shown as:

(3) $$IoU=\frac{Intersection}{Union}.$$

- Precision is defined as the ratio of true detected items in all the detected items. Let True denote the true classification and Positive denote detected. The mathematic form is shown as:

(4) $$Precision=\frac{TruePositive}{TruePositive+FalsePositive}$$

- Recall is defined as the ratio of true detected items in all the items that should be detected. The mathematic form is shown as:

(5) $$Recall=\frac{TruePositive}{TruePositive+FalseNegative}$$

- Average Precision (*AP*) is defined as the average of the average precision value when the recall value ranging from 0 to 1.00 by 0.01 with ten different thresholds for the *IoU* (Intersection over Union) ranging from 0.5 to 0.95 by 0.05. The mathematic form is shown as:

(6) $$AP=\frac{1}{10}\underset{\begin{array}{c} conf=0.5,\;\\ 0.55\ldots 0.95 \end{array}}{\sum }\frac{1}{100}\underset{\begin{array}{c} r=0,0.01\\ \ldots 1.00 \end{array}}{\sum }Precision\left(recall\right).$$

where *r* denotes the recall value and conf denotes the threshold set.

- *AP^S^*, *AP^M^*, and *AP^L^* are defined the same as *AP*, while only accounting for objects of fixed size including Small (denoted as *S*), Medium (denoted as *M*), and Large (denoted as *L*) instead of all objects detected. The mathematic form is shown as:

(7) $$AP^{t}=\frac{1}{100}\underset{\begin{array}{c} r^{t}=0,0.01\\ \ldots 1.00 \end{array}}{\sum }Precision^{t}\left(recall^{t}\right),t\in \left\{S,\;M,\;L\right\}$$

where *t* denotes the type of objects considered and *r^t^* denotes the recall value of *t* types of objects.

- *AP*^50^, and *AP*^75^ are defined the same as *AP*, while only taking the threshold of 0.5 and 0.75 into account instead of averaging the corresponding value of threshold from 0.5 to 0.95. The mathematic form is shown as:

(8) $$AP^{c}=\frac{1}{100}\underset{\begin{array}{c} r^{c}=0,0.01\\ \ldots 1.00 \end{array}}{\sum }Precision^{c}\left(recall^{c}\right),c\in \left\{50,75\right\}$$

where *c* denotes the specific threshold set and *r^c^* denotes the corresponding recall value.

- Accuracy is defined as the percentage of correctly predicted samples among the whole sample set. The mathematic form is shown as:

(9) $$Accuracy=\frac{correct\;prediction}{all\;samples}.$$

#Params is the whole set of hyper-parameters for the model, which can be used to evaluate the complexity and performance of the model from a real-time perspective.

### 4.2. Detection

In this part, the dataset and hyper-parameters used in the traffic sign detection are presented as well as the performance of each model.

#### 4.2.1. Experiment Description

The traffic sign detection task requires the detection of the location of the traffic sign in the picture, while we simultaneously detect and classify the location. The dataset we selected is the ITSDB dataset for traffic sign detection under the ice and snow environment, and we chose the Libra-RCNN [8] network proposed by CVPR in 2019, which obtained the best results in COCO and the best performance in ImageNet, and the HRNetv2p-w18 detection network [7], which is also one of the advanced networks to test on our dataset to verify the robustness and our dataset’s challenge.

We trained the model on the ITSDB of the original network for a fair comparison. We used Faster-RCNN as the baseline, set the model training epoch to 12, and set the batch size to 1. The initial learning rate was 0.025, and, when the epoch was 8 and 11, it decreased ten times and compared when the threshold was 0.5.

#### 4.2.2. Performance Comparison

**Figure 6:**
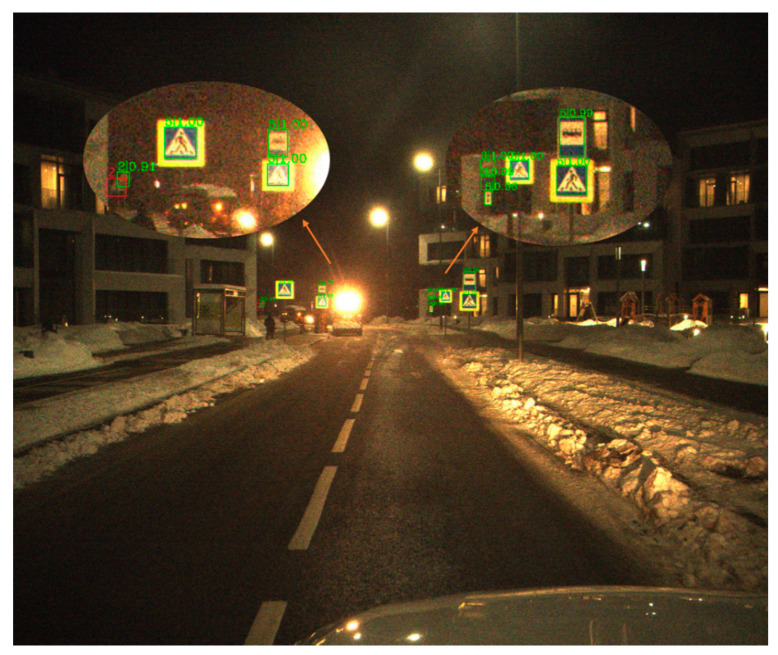
The visualization of the Libra-RCNN result on ITSDB detection. Green square represents detection result of the model, and red square represents target missed or wrong detection results.

**Figure 7:**
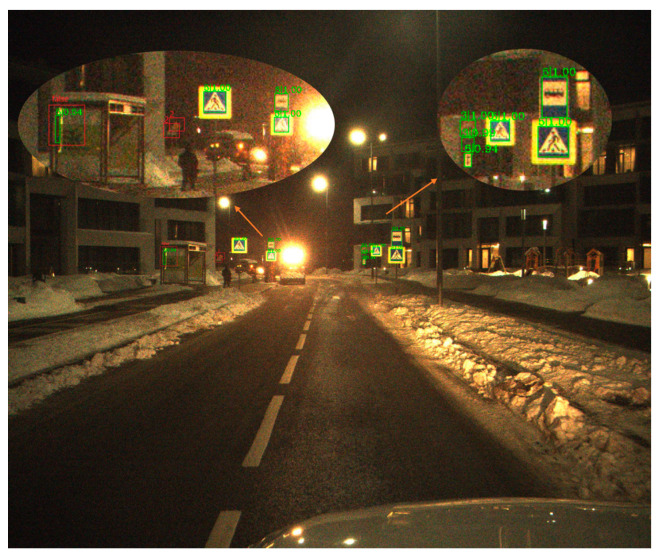
The visualization of the Faster-RCNN result on ITSDB detection. Green square represents detection result of the model, and red square represents target missed or detected wrong.

**Figure 8:**
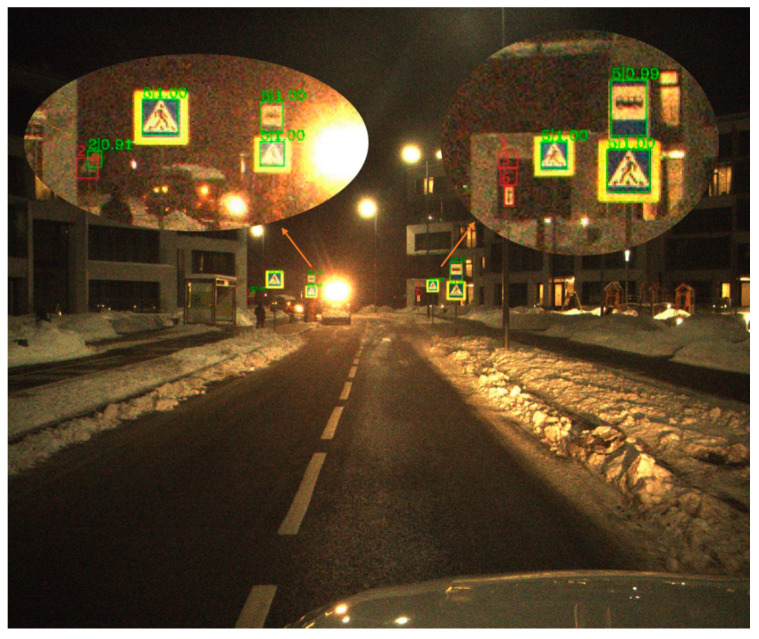
The visualization of the HRNet result on ITSDB detection. Green square represents detection result of the model, and red square represents target missed or detected wrong.

Table 2 shows the different types of Average Precision of three classical methods on ITSDB. Figure 6, Figure 7 and Figure 8 shows a visualization of the detection results of methods.

### 4.3. Classification

In this part, we present the dataset and hyper-parameters used in the traffic sign recognition as well as the performance of each model with detailed tables and figures.

#### 4.3.1. Experiment Description

The traffic sign recognition task requires accurate identification of the type of each traffic sign. In the common public dataset, GTSRB under a common environment, the category, train, contains 39,209 pictures, val contains 12,630 pictures, and each contains 43 categories. In the experiment, we trained our model both on our proposed dataset ITSRB and the public dataset GTSRB, and compared with other traffic sign classification networks. To make a fair comparison, we used single-crop testing in all tests, with Accuracy as the evaluation indicator.

We used two NVIDIA-P40 GPUs for training and testing, resized the dataset image to 224 × 224, made the normalization, and trained 60 epochs with a batch size of 32 × 2. The initial learning rate was 0.05, and it decreased by 10 times when the epoch was 30. The weight decay of the stochastic gradient descending parameter (SGD) was 0.0001, and the momentum was 0.9. We tested on the proposed ITSRB and GTSRB, and designed an ablation experiment on the public dataset GTSRB, which has a larger amount of data and more categories, challenging the classification ability more.

#### 4.3.2. Performance Comparison

Following the above experiment settings, we obtained the results of our proposed PFANet and other advanced methods on ITSRB and GTSRB. The results are shown in Table 3 and Table 4. The PFANet we proposed performed the best in the ITSRB with an Accuracy of 93.57%—the highest among the four methods—and had the best performance in the public dataset GTSRB with an accuracy of 97.21%.

**Figure 9:**
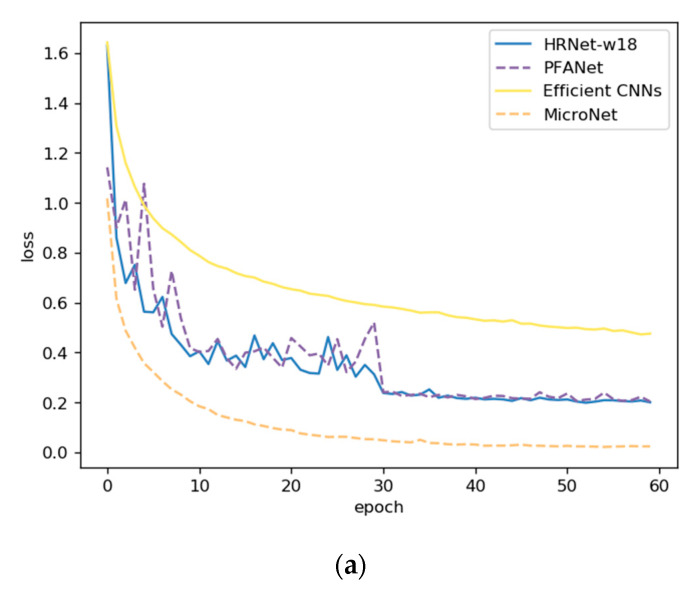

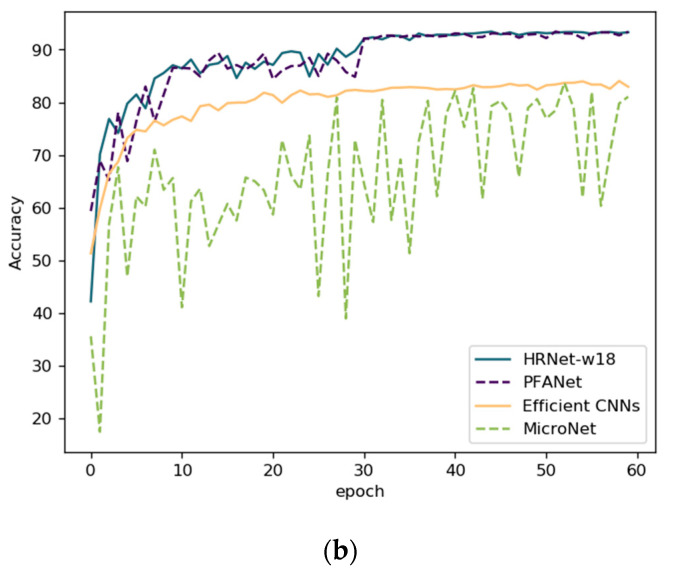
(**a**) The Training Loss curves on ITSRB. (**b**) The Training Accuracy curves on ITSRB.

**Figure 10:**
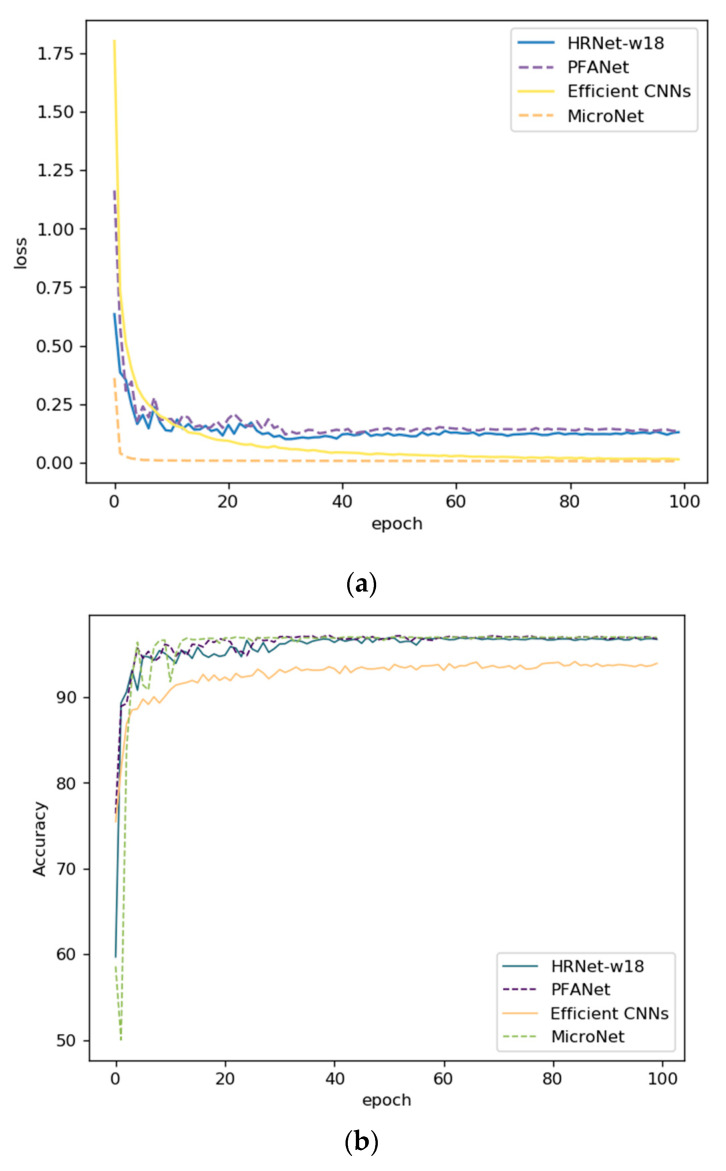
(**a**) The Training Loss curves on GTSRB. (**b**) The Training Accuracy curves on GTSRB.

The experimental details are shown in Figure 9 and Figure 10, including the loss curve and accuracy curve. PFANet had a higher initial accuracy and lower loss compared with the HRNetv2p-w18 and Efficient CNNs. Before the 30th epoch, PFANet oscillated more than the HRNet and Efficient CNNs in both loss and accuracy but also obtained better performance. After the 30th epoch, the oscillation was small, and it converged fast with a small learning rate. PFANet had the best performance model between the 50th and 60th epoch in ITSRB.

### 4.4. Ablation Study

In the experiments, we also performed a necessary ablation study to verify the modules we designed and applied in the PFANet. We followed the pipeline where each time we added one module into the network and tested its contribution to the whole performance, and compared its accuracy, GFLOPs and parameters with the baseline.

To verify the robustness, we ran the experiments on the widely-used open traffic dataset GTSRB. The input size was set to 224 × 224, the training epoch was 100, and the learning rate was 0.05 decreased by 10 times when the epoch was 30, 60, 90.

The results are shown in Table 5. Compared with the baseline, both the PFAN-A and PFAN-B module improved the performance, separately increasing the accuracy by 0.286 and 0.216. The proposed PFANet reached the state-of-the-art obtaining 97.213% accuracy. Compared with the results on ITSRB, all the networks had higher performance, implying the benchmark we made deserves more and deeper research, due to the lack of the environment and size complexity etc. Both attention modules increased the computation and parameters slightly. Both increased the parameters of 2.2 M, and PFAN-B increased the GFLOPs by 0.57, while the PFAN-B increased the GFLOPs by 0.43, lower than PFAN-B.

**Figure 11:**
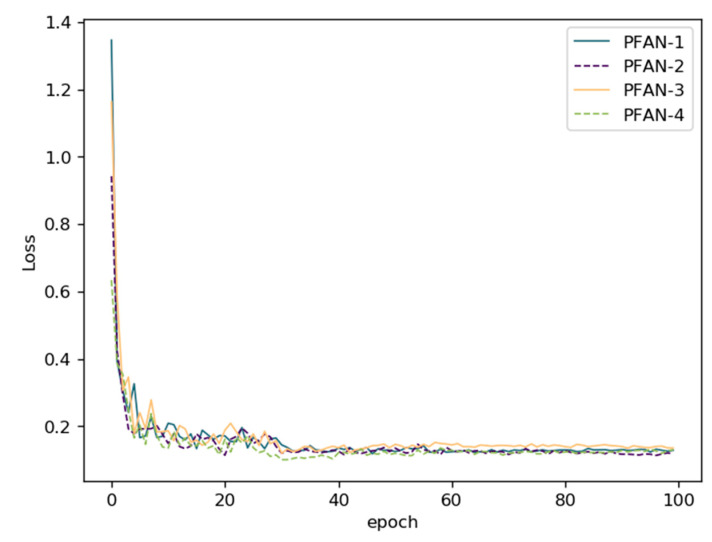
The training loss curves on GTSRB in the ablation study.

**Figure 12:**
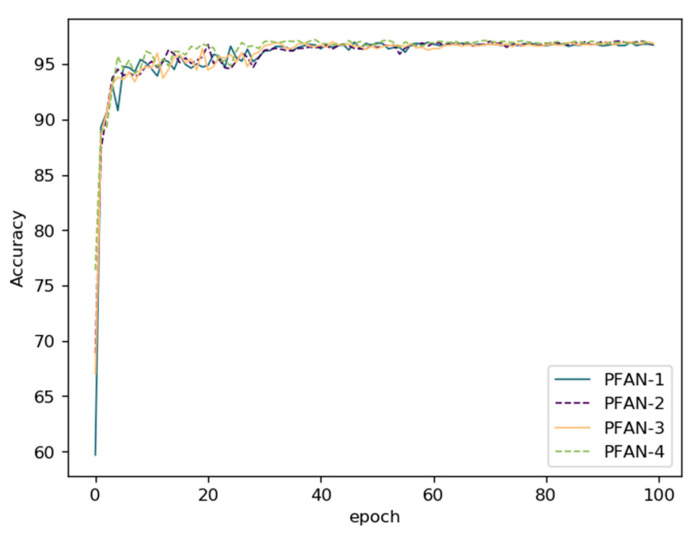
The training accuracy curves on GTSRB in the ablation study.

The loss curve and accuracy curve during training are drawn in Figure 11 and Figure 12. From the curves, both the PFAN-A and PFAN-B module could accelerate the convergence and obtain higher accuracy earlier than the baseline on GTSRB.

## 5. Discussion

First, from the results in Section 4.2, Libra-RCNN’s unbalanced design allowed it to perform better under small targets and high *IoU* requirements, acquiring the highest *AP*^75^, *AP*^S^, and *AP*^L^, while HRNet presented worst performance in all metrics on ITRDB. Under same computation amount, HRNet performed even worse than the Fast-RCNN. There might be two main reasons for this. First, HRNet may not overcome the unbalanced dataset in terms of category and size. It mainly takes the feature level unbalance into consideration and improves it by proposed multi-scale fusion. Second, under the same parameters, HRNet may be limited to find both useful bounding boxes prediction and classification features, especially in small target detection even with occlusion and light problems.

However, the Libra-RCNN proposed *IoU*-balanced sampling, balanced feature pyramid, and balanced L1 loss, overcoming the unbalance problem in data, objective, and feature level; therefore, it had better robustness under the ITSDB environment light factor and another complex, small target majority dataset. Compared with these models’ performance in their original papers in the COCO dataset, which is a larger dataset compared with ITSDB [16,17], they did not show any improvement. Therefore, this reveals that the ITSDB benchmark that we proposed is suitable for the robustness evaluation of models from the unbalance, which is quite close to reality.

Discussion regarding the main contribution, the attention modules and network we proposed in this paper based on the ablation study in the working mechanism and module comparison is presented as follows. PFAN-A is designed to highlight the underlying information in a feature map, which may be helpful for classification, especially under snow and ice environments. The design of PFAN-B compares the feature information between different feature maps by querying to highlight the intersection between different resolution feature maps of the same picture information. This part of the information is deep attribute information after extracting different semantic information and external information, and this is the main category information for traffic signs.

We hope to solve the information interference caused by the environment, time, light, occlusion, etc. in ice and snow environments through this design to find and pay attention to the most effective information in the picture and solve the information coverage and fusion caused by the pure fusion of different resolution maps at the baseline. Our comprehensive design was able to learn deep feature representations under multiple interferences.

As shown in Table 4, the PFAN network with one of these structures added alone had only a slight improvement over the baseline. For PFAN-1, the main reason is that direct fusion leads to PFAN-A highlighting the weight of possible favorable classification information in a single feature map, which is diluted in the fusion by the feature information of another feature map that is not highlighted, thereby, reducing the effect but still remaining slightly better than PFAN-2. For PFAN-2, the main reason is that direct convolution does not pre-select possible advantageous features, resulting in PFAN-B only exerting the effect of increasing the network depth.

Compared with the baseline, PFANet improved the accuracy by 0.4 while increasing PFAN-A and PFAN-B. This confirms that repeating the structure of pre-selecting feature information through PFAN-A, selecting feature information through PFAN-B cross-comparison, and then fusing can produce more effective classification information. Therefore, we added both modules in the final network, which indeed learned deep feature representations under multiple interferences under a snow and ice environment.

In Section 4.3, from the two line-charts, PFANet oscillated more than the other models. From the previous studies, this may be caused by the extra computation added by the attention modules. When optimizing the result, the extra paraments required optimization and differed a great deal. After 30 epochs, both converged, and PFANet reached higher performance by finding the essential features even in the ITSRB, showing outstanding robustness. However, the previous models, Efficient CNN and MicroNet, only had accuracies lower than 85%, and MicroNet even had severe oscillation in ISTRB. This is because their networks are weak in extracting useful features under the effects of the environment. PFANet also achieved the highest accuracy in GTSRB, whose images were collected under a normal environment, intuitively showing its robustness.

Comparing the results on the ITSRB and GTSRB, all the networks reached lower accuracy than on GTSRB as there were more occluded or including environment factors in the images compared with in the benchmark ITSRB, although both show some robustness. This also reveals, from another angle, that our ITSRB has greater challenges and can be used to train models overcoming ice environment influences applied in real time self-driving.

In the future, we will investigate the main reason for the oscillation of PFANet at the beginning, attempt to decrease the parameters to obtain a smaller network, and focus on improving the performance of recognition under further special environments in addition to light, occlusion, etc. with advanced mechanisms. We hope to apply these findings in automobiles to improve their intelligence.

## 6. Conclusions

In this article, we proposed a new traffic sign detection dataset (ITSDB) under an ice and snow environment and a new traffic sign classification dataset (ITSRB) under an ice and snow environment. Compared with the previous dataset, our dataset contains pictures of different sizes, ice and snow environments, and occlusion, with greater flexibility and presents a greater challenge for traffic sign recognition. We also put forward a parallel attention network, PFANet, which can extract more effective features through the combination of two attention modules, PFAN-A and PFAN-B, to overcome recognition difficulties caused by the complex factors in the ice and snow environment.

PFANet achieved the best performance of 93.570% accuracy on ITSRB and 97.21% on GTSRB without data augmentation, which verifies our design concept. We trained two of the newest networks that simultaneously detect and classify on ITSDB and found that Libra-RCNN was more robust. We proposed possible reasons for this in the discussion. In the future, we will continue to explore in-depth automated traffic sign recognition methods.

## Figures and Tables

**Table 1:** The quantity of icons of different sizes in the dataset.

Size Category	Size	ITSDB (Ours)	GTSDB [2]
Small	$size\leq 32^{2}$	28,487 (**65.8%**)	8778 (16.9%)
Medium	$32^{2}\leq size\leq 96^{2}$	11,916 (27.5%)	40,091 (**77.3%**)
Large	$\;size\geq 96^{2}$	2887 (3.7%)	2970 (5.7%)

**Table 2:** Test results on ice environment traffic sign detection benchmark (ITSDB) with HRNet and Libra-RCNN under the Faster-RCNN architecture.

	Backbone	*AP*	*AP* ^50^	*AP* ^75^	*AP* ^S^	*AP* ^M^	*AP* ^L^
Faster-RCNN [32]	Resnet-50	46.5	**66.6**	53.8	33.1	**71.5**	89.3
HRNetv2p-w18 [16]	-	29.8	40.3	33.6	11.5	65.0	87.7
Libra-RCNN [17]	Resnet-50	**47.0**	64.4	**54.8**	**34.0**	70.7	**90.4**

**Table 3:** The recognition results on ITSRB (proposed dataset).

Method	#Params.	Data Augmentation	Input Size	Epoch	Accuracy
HRNet-w18 [16]	19.3 M	×	224 × 224 × 3	60	93.42
EfficientNet [14]	0.95 M	×	48 × 48 × 3	60	84.04
MicroNet [13]	0.51 M	×	48 × 48 × 3	60	83.72
PFANet (ours)	27.6 M	×	224 × 224 × 3	60	**93.57**

**Table 4:** The recognition results on German traffic sign recognition dataset (GTSRB) (public dataset). “*” represents result of the method is drawn from the literature, and without “*” represents the result is obtained from the re-trained model.

Method	#Params.	Data Augmentation	Input Size	Epoch	Accuracy
sermanet * [11]	×	√	32 × 32 × 3	×	99.17
IDSIA * [10]	×	√	48 × 48 × 3	×	**99.46**
HRNet-w18 [16]	19.3 M	×	224 × 224 × 3	100	96.80
EfficientNet [14]	0.95 M	×	48 × 48 × 3	100	94.14
MicroNet [13]	0.51 M	×	48 × 48 × 3	100	97.02
PFANet (ours)	27.6 M	×	224 × 224 × 3	100	**97.21**

**Table 5:** The ablation experiment results of the proposed modules on the GTSRB val dataset, PFAN-A stands for the PFAN-A module, PFAN-B stands for the PFAN-B module, PFAN-1 stands for the baseline, and PFAN-4 stands for the proposed PFANet.

Method	PFAN-A	PFAN-B	#Params.	GFLOPs	Accuracy
HRNet [16]	×	×	21.3 M	3.99	96.80
PFAN-2	√	×	23.5 M	4.56	97.09
PFAN-3	×	√	23.5 M	4.42	97.02
PFANet (ours)	√	√	27.7 M	4.99	**97.21**

## Data Availability

The data presented in this study are available on request from the second or corresponding author. The data are not publicly available due to further project application.
